# A Rice Plastidial Nucleotide Sugar Epimerase Is Involved in Galactolipid Biosynthesis and Improves Photosynthetic Efficiency

**DOI:** 10.1371/journal.pgen.1002196

**Published:** 2011-07-28

**Authors:** Chunlai Li, Yiqin Wang, Linchuan Liu, Yingchun Hu, Fengxia Zhang, Sod Mergen, Guodong Wang, Michael R. Schläppi, Chengcai Chu

**Affiliations:** 1 State Key Laboratory of Plant Genomics and National Center for Plant Gene Research, Institute of Genetics and Developmental Biology, Chinese Academy of Sciences, Beijing, China; 2 Graduate University of the Chinese Academy of Sciences, Beijing, China; 3 College of Life Sciences, Peking University, Beijing, China; 4 Department of Biological Sciences, Marquette University, Milwaukee, Wisconsin, United States of America; Peking University, China

## Abstract

Photosynthesis is the final determinator for crop yield. To gain insight into genes controlling photosynthetic capacity, we selected from our large T-DNA mutant population a rice stunted growth mutant with decreased carbon assimilate and yield production named *photoassimilate defective1* (*phd1*). Molecular and biochemical analyses revealed that *PHD1* encodes a novel chloroplast-localized UDP-glucose epimerase (UGE), which is conserved in the plant kingdom. The chloroplast localization of PHD1 was confirmed by immunoblots, immunocytochemistry, and UGE activity in isolated chloroplasts, which was approximately 50% lower in the *phd1*-*1* mutant than in the wild type. In addition, the amounts of UDP-glucose and UDP-galactose substrates in chloroplasts were significantly higher and lower, respectively, indicating that PHD1 was responsible for a major part of UGE activity in plastids. The relative amount of monogalactosyldiacylglycerol (MGDG), a major chloroplast membrane galactolipid, was decreased in the mutant, while the digalactosyldiacylglycerol (DGDG) amount was not significantly altered, suggesting that *PHD1* participates mainly in UDP-galactose supply for MGDG biosynthesis in chloroplasts. The *phd1* mutant showed decreased chlorophyll content, photosynthetic activity, and altered chloroplast ultrastructure, suggesting that a correct amount of galactoglycerolipids and the ratio of glycolipids versus phospholipids are necessary for proper chloroplast function. Downregulated expression of starch biosynthesis genes and upregulated expression of sucrose cleavage genes might be a result of reduced photosynthetic activity and account for the decreased starch and sucrose levels seen in *phd1* leaves. *PHD1* overexpression increased photosynthetic efficiency, biomass, and grain production, suggesting that PHD1 plays an important role in supplying sufficient galactolipids to thylakoid membranes for proper chloroplast biogenesis and photosynthetic activity. These findings will be useful for improving crop yields and for bioenergy crop engineering.

## Author Summary

Photosynthesis is carried out in chloroplast, a plant-specific organelle. Photosynthetic membranes in chloroplasts contain high levels of glycolipids, and UDP-galactose is a dominating donor for glycolipid biosynthesis. Although glycolipid assembly of photosynthetic membranes has been characterized at the genetic and enzymatic level, the mechanism of substrate supply of UDP-galactose for the glycolipid biosynthetic pathway remains obscure. By genetic screening of rice mutants that are impaired in photosynthetic capacity and carbon assimilation, we identified PHD1 as a novel nucleotide sugar epimerase involved in a process of glycolipid biosynthesis and participating in photosynthetic membrane biogenesis. *PHD1* was preferentially expressed in green and meristem tissues, and the PHD1 protein was targeted to chloroplasts. We revealed that UDP-galactose for glycolipid biosynthesis catalyzed by the new enzyme was generated inside chloroplasts, and the reduced amounts of glycolipids in the mutant led to decreased chlorophyll content and photosynthetic activity. Overexpression of this gene lead to growth acceleration, enhanced photosynthetic efficiency, and finally improved biomass and grain yield in rice. These results suggest that PHD1 has significant economic implications in both traditional crop improvement and bioenergy crop production.

## Introduction

Plants possess a sophisticated sugar biosynthetic machinery comprised of families of nucleotide sugars that can be modified at their glycosyl moieties by nucleotide sugar interconversion enzymes to generate different sugars [1], [2]. UDP-glucose 4-epimerase (also UDP-galactose 4-epimerase, UGE; EC 5.1.3.2) catalyzes the interconversion of UDP-D-glucose (UDP-Glc) and UDP-D-galactose (UDP-Gal) [3], [4]. UGE is essential for *de novo* biosynthesis of UDP-Gal, a precursor for the biosynthesis of different carbohydrates, glycolipids, and glycosides. Genes encoding UGE have been cloned from a range of different organisms including bacteria, yeast, and human [5]–[7], and the crystal structures have also been obtained [8]–[10].

The original biochemical and genetic analyses of UGE in plants was described by Dörman and Benning [11]. To date, five UGE isoforms have been identified in Arabidopsis [2], [12], three in barley [13], and a family of four putative UGE isoforms exist in rice. In Arabidopsis, global co-expression analysis revealed that *UGE2*, -*4*, and -*5* preferentially act in the UDP-Glc to UDP-Gal directions, whereas *UGE1* and *UGE3* might act in the UDP-Gal to UDP-Glc directions [14]. Reverse genetic studies demonstrated that *UGE2* and *UGE4* influence vegetative growth and cell wall carbohydrate biosynthesis, that *UGE3* is specific for pollen development, and that *UGE1* and *UGE5* act in stress situations [15], [16]. Compared to 4-day-old seedlings, *UGE* expression increased 5-fold in roots of 3-week-old pea plants, suggesting that increased *UGE* expression correlated with the copious secretion of pectinaceous mucigel in older seedling roots [17]. To date, all UGEs identified from plants lack transmembrane motifs and signal peptides and appear to exist as soluble entities in the cytoplasm.

Photosynthetic reactions in higher plants depend on the well-developed chloroplast thylakoid membrane system. Chloroplast thylakoid assembly and maintenance require a continuous supply of membrane constituents. Galactose-containing glycerolipids are predominant lipid components of photosynthetic membranes in plants, algae, and cyanobacteria. The two most common galactolipids are mono- and digalactosyldiacylglycerol (MGDG and DGDG), which account for about 50 and 25 mol% of total thylakoid lipids, respectively [18], [19]. About 80% of all plant lipids are associated with photosynthetic membranes, and MGDG is considered to be the most abundant membrane lipid on earth. Recent studies have demonstrated that galactolipids play an important role in not only the organization of photosynthetic membranes, but also in their photosynthetic activities [20], [21]. Arabidopsis mutants with a lower amount of these galactolipids have a reduction in chlorophyll content and photosynthetic activity, alterations in chloroplast ultrastructure, and impairment of growth [22]–[25].

In plants, MGDG is synthesized in two unique steps: (i) the conversion of UDP-Glc into UDP-Gal by an UGE, and (ii) the transfer of a galactosyl residue from UDP-Gal to diacylglycerol (DAG) for synthesis of the final product by MGDG synthase (MGD1) [26], [27]. Although MGD1 has been characterized at both genetic and enzymatic levels, the UDP-Gal supply mechanisms for the MGDG biosynthetic pathway remain obscure. MGD1 is localized in the inner chloroplast envelope membrane [26], [27] and uses UDP-Gal as a substrate. However, the concentration of UDP-Gal in chloroplasts is considered to be very low [28], suggesting that the UDP-Gal source is imported from the cytosol or generated inside chloroplasts.

To gain insight into genes controlling photosynthetic activity and carbon assimilation in plants, a rice stunted growth mutant (*phd1*) with decreased photoassimilate and yield production was selected for further study from a large-scale screening of our T-DNA mutant population. Interestingly, *PHD1* encoded a chloroplast-localized UDP-Glc epimerase involved in UDP-Gal supply for chloroplast galactolipid biosynthesis during photosynthetic membrane biogenesis. Its homologs are highly conserved in the plant kingdom, and the gene was preferentially expressed in various young meristems where plastid proliferation actively occurred. Most strikingly, overexpression of *PHD1* increased photosynthetic activity and enhanced rice growth. The important roles of PHD1 in photosynthetic capability and carbon assimilate homeostasis are discussed.

## Results

### Isolation and characterization of the *phd1* mutant

**Figure 1:**
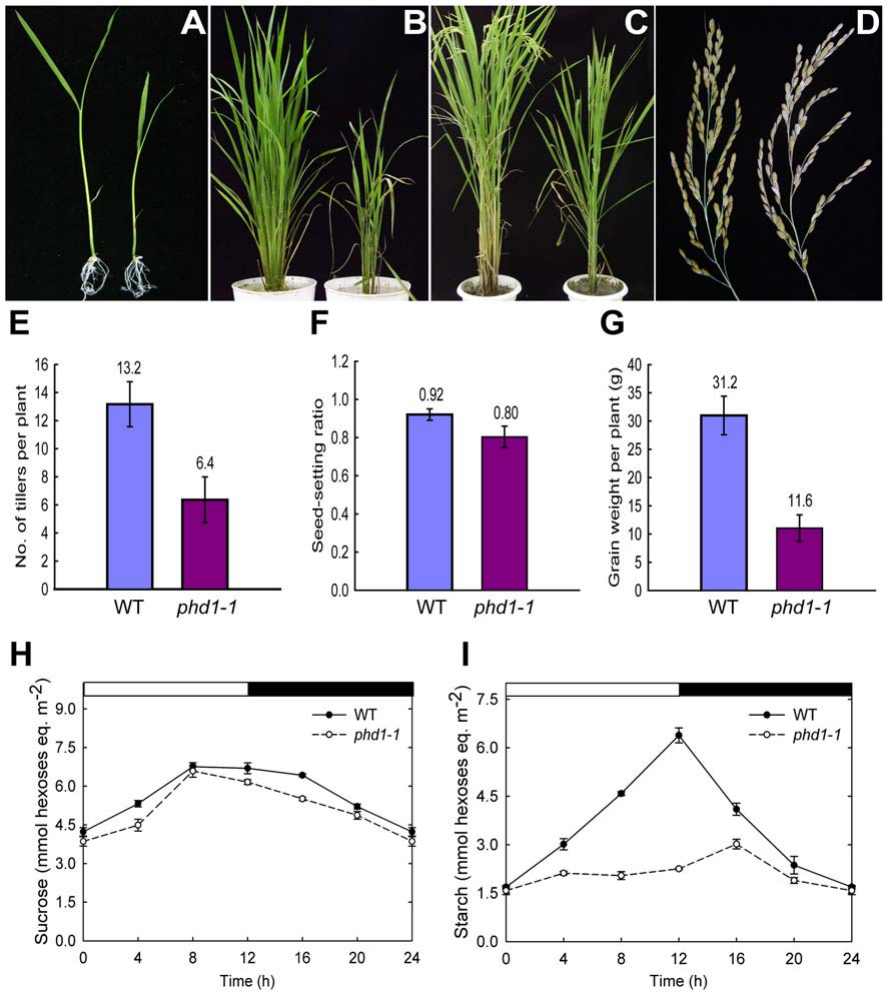
*phd1* mutant phenotypes. (A) Two-week-old seedlings grown on MS medium. (B and C) Growth phenotype of 2-month-old (B) and 4-month-old (C) plants grown in a paddy field. (D) The harvested panicles showed a reduced seed-setting ratio for *phd1*-*1*. (A–D) wild type (left) and *phd1*-*1* (right). (E–G) Quantification of the agricultural traits of tiller number (E), seed setting ratio (F), and grain weight per plant (G). Each bar is the mean ± SD from 30 replicate samples. (H, I) Diurnal changes in sucrose (H) and starch (I) content of *phd1*-*1* and wild type. Mature leaves of individual wild type and *phd1-1* plants at the anthesis stage were harvested and immediately frozen in liquid nitrogen. Each point is the mean ± SD from ten replicate samples.

To identify genes affecting photosynthetic activity and carbon assimilation, a large-scale screening of our rice T-DNA insertion mutant population (*Oryza sativa* var. Nipponbare background) [29] was carried out. Of 480 mutant lines with altered carbohydrate levels in vegetative organs, *photoassimilate defective1* (*phd1*) with both low carbohydrate contents and stunted growth was selected for further characterization. Scanning electron micrograph of culms demonstrated that fewer starch granules were deposited in parenchyma cells of the *phd1* mutants (data not shown). During the young seedling stage, both shoots and primary roots of the mutant were shorter and lighter than those of the wild type (Figure 1A). After internode elongation, the *phd1* mutant exhibited a semi-dwarf, less grain-filling, retarded vegetative growth, later flowering, and less tillering phenotype (Figure 1B–1E). In addition, although the grain number per panicle was not altered between the mutant and wild type, the seed-setting ratio of the *phd1* mutant was significantly decreased, which finally led to a significant reduction of grain yield (Figure 1F, 1G). Compared to wild type, mature leaves of the mutant had somewhat reduced sucrose (Figure 1H) and rather low starch levels (Figure 1I) at all time-points taken during the light/dark cycle, while hexose levels were a little higher in the mutant (Figure S1).

### *PHD1* encodes a functional UDP-Glc epimerase

**Figure 2:**
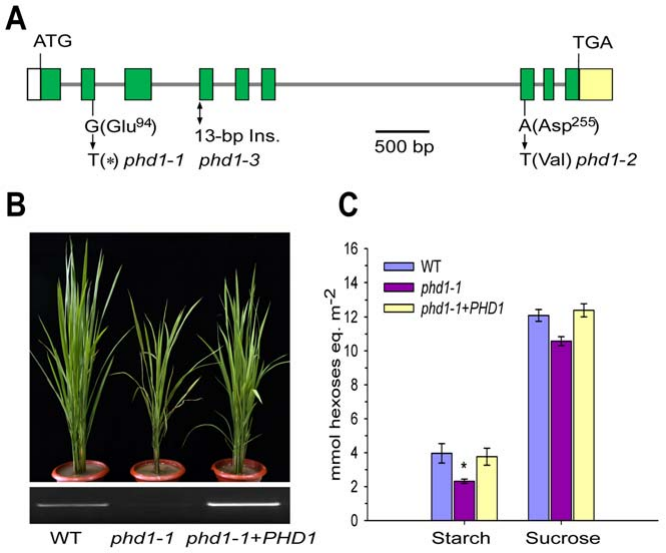
Molecular identification of *PHD1*. (A) Structure of the *PHD1* gene and its mutation sites in three *phd1* alleles. The *PHD1* gene consists of nine exons (green boxes) and eight introns (gray lines). Nucleotide insertion and substitutions in the three *phd1* alleles are indicated. (B, C) Functional complementation of the *phd1* mutant. (B) Upper panel: Phenotypes of wild type, *phd1*-*1*, and complemented *phd1*-*1*+*PHD1* plants at the tillering stage. Lower panel: Expression levels of *PHD1* transcripts as detected by semi-quantitative RT-PCR. (C) Sucrose and starch content in flag leaves of wild type, *phd1*-*1*, and complemented *phd1*-*1*+*PHD1* plants at noon of the day at the anthesis stage. Error bars represent SD of eight different individuals. *significant difference between *phd1*-*1* mutant and wild type (*P* = 0.05).

Genetic analysis indicated that the *phd1* phenotype was controlled by a single recessive gene that did not co-segregate with the T-DNA insertion, and hence map-based cloning was carried out. The *PHD1* locus was physically delimited to a 72-kb region on the short arm of chromosome 1. This region contains six annotated genes, and sequencing of these genes from *phd1*-*1* identified a single nucleotide transition (G-to-T) in exon 2 of *Os01g0367100*, leading to a premature translational termination. The identity of *Os01g0367100* as *PHD1* was confirmed by analysis of two other *phd1* alleles with similar phenotypes isolated from the same genetic screen, for which a single nucleotide substitution (A-to-T) in exon 7 in *phd1*-*2* and a 13-bp insertion between exon 3 and exon 4 in *phd1*-*3* were found (Figure 2A). Almost no *PHD1* mRNA was detected in any of the three allelic mutants (Figure S2). The *phd1* phenotype was complemented by transgenic expression of wild type *Os01g0367100* in the *phd1*-*1* mutant background (Figure 2B, 2C), confirming that the nonsense mutation of *Os01g0367100* was responsible for the presumed null mutant phenotype.

**Figure 3:**
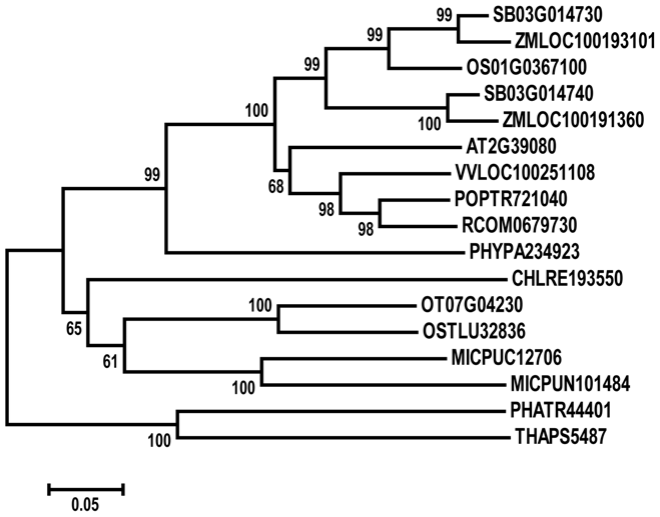
Phylogenetic analysis of PHD1. MEGA4 Neighbor-Joining tree was inferred from the amino acid sequences of the PHD1 (Os01g0367100) homologs among green plants. Bootstrap values are based on 1 000 replications and are indicated in their respective nodes. The scale bar indicates genetic distance based on branch length. An alignment for the constructed tree is shown in Figure S3.

Database searches revealed that PHD1 has similarity to proteins from *Thalassiosira pseudonana* (XP_002290295), *Phaeodactylum tricornutum* (XP_002178225), *Chlamydomonas reinhardtii* (XP_001699105), *Micromonas pusilla* (EEH60780), *Ostreococcus tauri* (CAL54696), *Physcomitrella patens* (XP_001767242), *Ricinus communis* (XP_002516868), *Arabidopsis thaliana* (AT2G39080), *Populus trichocarpa* (XP_002311843), *Vitis vinifera* (XP_002276706), *Zea mays* (NP_001131736), and *Sorghum bicolor* (XP_002457832), with 27 to 75% amino acid identity (Figure S3). Phylogenetic analysis between PHD1 and its 16 putative homologs indicated that PHD1 is closely related to Sb03g014730 from sorghum and LOC100193101 from maize (Figure 3). PHD1 homologs are only found in the plant kingdom, suggesting that these proteins are evolutionally conserved across plant species. However, none of the homologous genes have been functionally characterized. Analysis of the conserved domain demonstrated that PHD1 and its homologs contain the consensus WcaG domain, featured in nucleoside-diphosphate sugar epimerases (Figure S3). One of the best characterized nucleotide sugar epimerases is UDP-Glc epimerase, which catalyzes the interconversion of UDP-Glc and UDP-Gal. Hence, PHD1 and its homologs may function as novel plant specific UDP-Glc epimerases.

To validate PHD1's biochemical function as an UDP-Glc epimerase, the mature PHD1 protein lacking the putative N-terminal 62-aa transit peptide was expressed in *E. coli* and UGE activity was examined. The result showed that PHD1 could catalyze the conversion of UDP-Gal to UDP-Glc, and curve fitting indicated that UDP-Gal binding followed a simple Michaelis-Menten kinetics with a *K*_m_ value of 0.84 mM at 30°C (Figure S4A). To examine whether PHD1 had UDP-Glc epimerase activity *in vivo*, the mature PHD1 under the control of the yeast glyceraldehyde-3-phosphate dehydrogenase promoter was used to complement the auxotrophic phenotype of a yeast *gal10Δ* mutant which cannot grow on a medium containing D-galactose as sole carbon source. The complementation results demonstrated that PHD1 also had UDP-Glc epimerase activity *in vivo* (Figure S4B).

**Figure 4:**
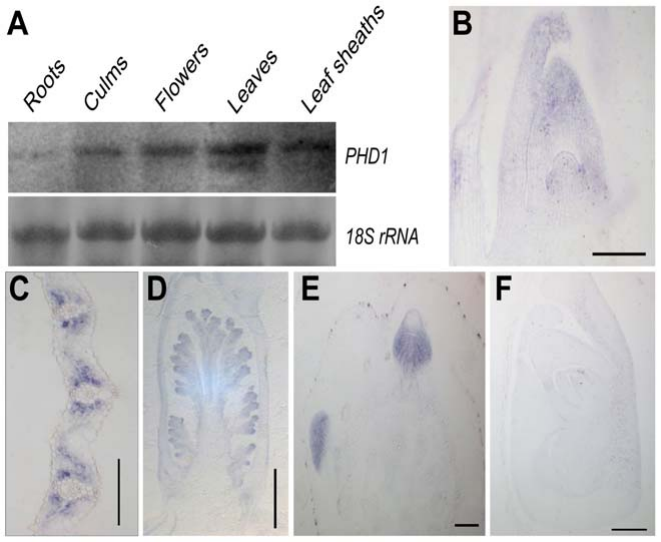
Expression analysis of *PHD1*. (A) RNA gel blot analysis of the *PHD1* gene in roots, culms, flowers, leaf blades, and leaf sheaths just before the anthesis stage. (B–F) *PHD1* expression patterns detected by mRNA *in situ* hybridization. The *PHD1* signal was detected in the shoot apical meristem and young leaves (B), leaf mesophyll cells around vascular bundles (C), young inflorescences (D), and axillary buds (E). (F) Negative control preparation made with a *PHD1* sense probe. Bars = 150 µm in (B), (C), (E), and (F), and 500 µm in (D).

RNA gel blot analysis revealed that *PHD1* was present in all green tissues, with highest abundance in leaf blades and leaf sheaths, then flowers and culms, but only at very low levels in roots (Figure 4A). mRNA *in situ* hybridization using an antisense probe revealed that *PHD1* was expressed predominantly in leaf primordia and shoot apical meristems (Figure 4B), the mesophyll cells surrounding the vascular bundles of young leaves (Figure 4C), inflorescence primordia (Figure 4D), and axillary buds (Figure 4E). In contrast, hybridization with a *PHD1* sense probe showed no signal (Figure 4F).

### PHD1 is targeted to the chloroplast

**Figure 5:**
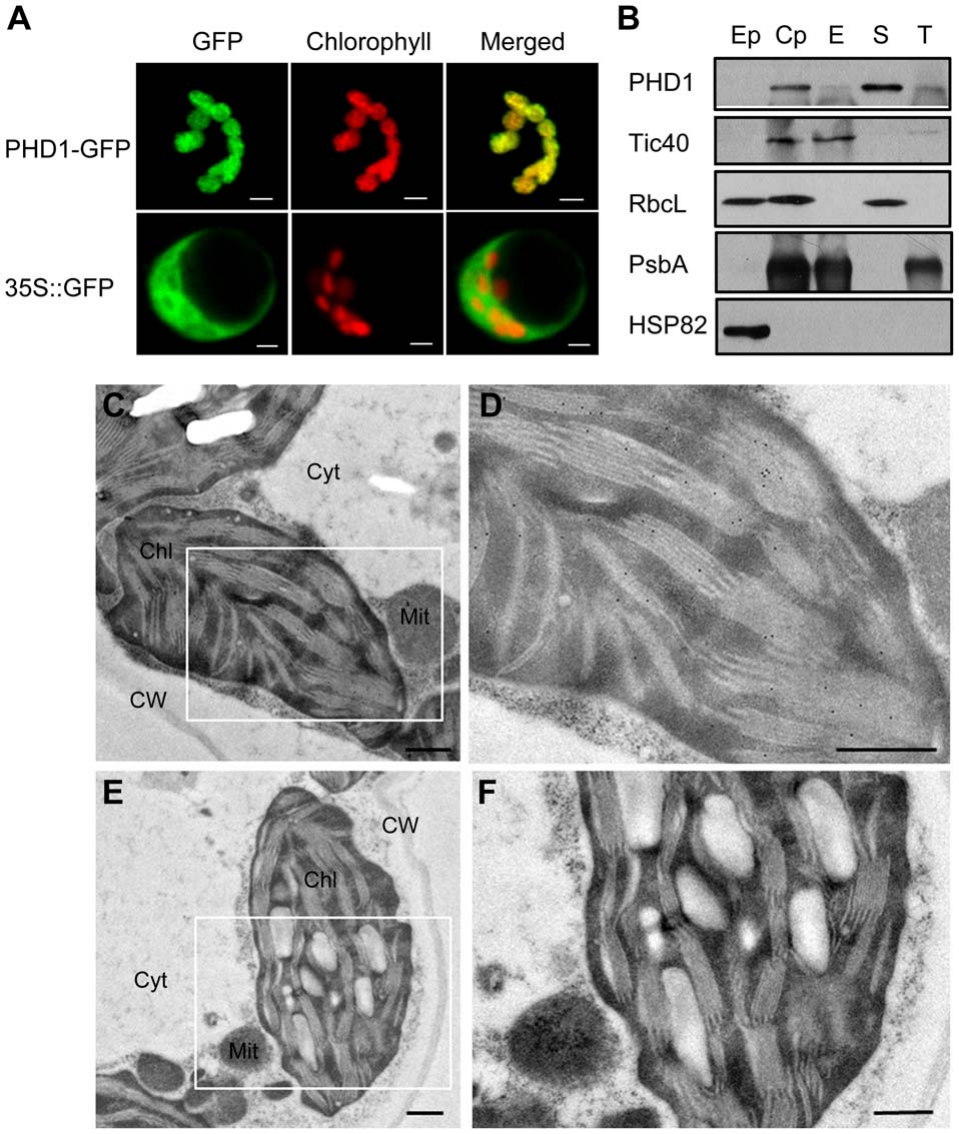
Subcellular localization of PHD1. (A) Confocal micrographs showing chloroplast targeting of PHD1. Rice protoplasts transformed with 35S::*PHD1*-GFP (upper panel) and 35S::GFP (lower panel) plasmids are shown. Chlorophyll autofluorescence (middle); GFP fluorescence (left); merged images (right). Bars = 5 µm. (B) PHD1 protein distribution in chloroplast subfractions. Percoll-purified intact chloroplasts were lysed and subjected to differential centrifugation fractionation into envelope, stroma, and thylakoid fractions. Proteins were separated by SDS-PAGE, and blotted against the PHD1 antibody and specific chloroplast subcompartment protein antibodies. Tic 40 is an envelope membrane protein, RbcL a stroma protein, and PsbA a thylakoid membrane protein. HSP82 was used as a cytosolic protein marker. About 15 µg of total proteins from extrachloroplast (Ep), purified chloroplast (Cp), envelope (E), stroma (S), and thylakoid (T) subfractions were loaded per line. RbcL seen in the Ep fraction is most likely due to leakage from the stroma of broken chloroplasts. (C–F) Immunogold localization of PHD1. Thin sections of chloroplasts in leaf mesophyll cells were incubated with PHD1 antibodies (C and D) and preimmune serum (E and F). The gold label is found preferentially associated with thylakoids of the chloroplasts as seen in (D). Chl, chloroplast; Cyt, cytosol; Mit, mitochondria; CW, cell wall. Bar = 0.5 µm.

*PHD1* encodes a 340 aa protein with a putative 62-aa chloroplast transit peptide at the N-terminus. To confirm chloroplast localization of PHD1, the full-length *PHD1* was fused to the green fluorescent protein (GFP) reporter gene under the control of the cauliflower mosaic virus (CaMV) 35S promoter and subsequently transformed into rice shoot protoplasts. Figure 5A shows that GFP fluorescence co-localized with the red chlorophyll autofluorescence, confirming that PHD1 was a chloroplast-localized protein and the predicted transit peptide was functional.

To further investigate the subcellular localization of PHD1, we performed western blot experiments using purified plastid subfractions (Figure 5B). Several antibodies were used as specific markers for the different chloroplast subfractions. Tic 40 was used as a specific envelope marker, and Rubisco, the major stroma protein, as a marker of this chloroplast subfraction. PsbA, one of the components of photosystem II (PSII), was used as a marker to validate the thylakoid membrane fraction, and HSP82 was used as a cytosol specific marker. As shown in Figure 5B, the PHD1 protein was detected mainly in the stroma fraction and was absent from the cytoplasmic compartment, thus confirming that PHD1 was a chloroplast-targeted protein. To complete the subcellular localization study and to obtain additional information about the distribution of PHD1 in different chloroplast subcompartments, we further performed immunocytochemical analysis on ultrathin sections of rice tissues using polyclonal PHD1 antiserum. The positive signal of PHD1, visualized as black dots, was found specifically in the chloroplasts (Figure 5C and 5D). In contrast, sections treated with a preimmune serum (Figure 5E and 5F) showed no signal. The overall data thus strongly indicated that PHD1 is targeted to chloroplasts in rice.

### UGE activity is severely reduced in chloroplasts isolated from the *phd1*-*1* mutant

**Figure 6:**
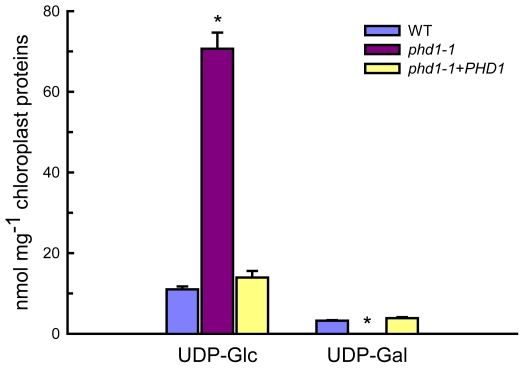
UDP-Glc and UDP-Gal levels in isolated chloroplasts from wild type, *phd1*-*1*, and *PHD1*-complemented transgenic lines. Intact chloroplasts were isolated from leaves of wild type, *phd1*-*1*, and *PHD1*-complemented plants by step-wise density gradient centrifugation, and UDP-sugar levels were determined as described in Materials and Methods. Values are the mean of three experiments ± SDs. Asterisks indicate a statistically significant difference from the wild type (*P<0.05, Student's *t*-test).

Intact chloroplasts were isolated from leaves of wild type and *phd1*-*1* mutant plants, and the UGE activity in isolated chloroplasts was measured (Figure S5). Compared to the wild type, a severe decrease (ca. 50%) in UGE activity was observed in isolated chloroplasts from the *phd1*-*1*mutant compared with the wild type, suggesting that PHD1 was responsible for a major part of the UGE activity in chloroplasts. Moreover, levels of the UGE substrates UDP-Glc and UDP-Gal in isolated chloroplasts were also determined (Figure 6). While compared to wild type and complemented mutant an overabundance of UDP-Glc was found in chloroplasts isolated from the *phd1*-*1* mutant, almost no amount of UDP-Gal was detected in the mutant. The levels of nucleotide sugars in whole leaves were also determined, which showed that the amount of UDP-Gal was slightly higher in *phd1*-*1* than in wild type plants, and the UDP-Glc amount was significantly higher (Figure S6). Hence, the ratio of UDP-Glc to UDP-Gal in *phd1*-*1* leaves was also higher than in wild type plants. These results suggested that *PHD1* dysfunction may trigger an accumulation of substrates and disturb the balance of interconversion between the two sugar nucleotides.

### *PHD1* dysfunction affects the photosynthetic membrane system

**Figure 7:**
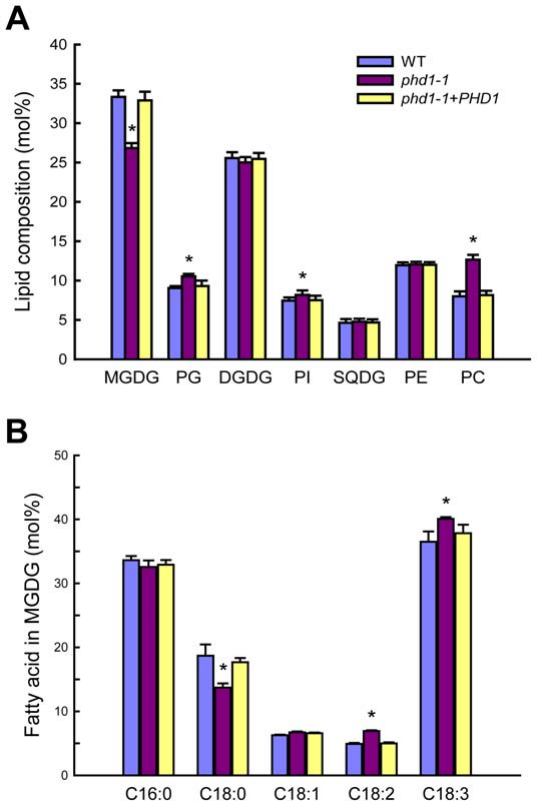
Polar lipid composition and fatty acid profiles in leaves of wild type, *phd1*-*1*, and *PHD1*-complemented plants. (A) Polar lipid composition in leaves of wild type, *phd1*-*1*, and *PHD1*-complemented plants grown in paddy fields. Glycerolipids were quantified by GC/MS of leaf lipids separated by TLC. The bars show lipid composition in mol% and indicate means ± SD of three measurements. PC, phosphatidylcholine; PE, phosphatidylethanolamine; PG, phosphatidylglycerol; PI, phosphatidylinositol; SQDG, sulfoquinovosyldiacylglycerol. (B) Fatty acid composition of MGDG from wild type, *phd1*-*1*, and *PHD1*-complemented plant leaves. The data are presented as the average mole percent of fatty acid for the indicated fatty acids along the x axis. The error bars represent the standard deviations of three biological replicates. Asterisks indicate a statistically significant difference from the wild type (*P<0.05, Student's *t*-test).

Chloroplast membranes contain high levels of glycolipids, and UDP-Gal is a dominant substrate for glycolipid biosynthesis. To examine the effect of *PHD1* dysfunction on membrane lipid homeostasis, the composition of total lipids extracted from *phd1*-*1*, wild type, and *PHD1*-complemented plants was analyzed (Figure 7). In the *phd1*-*1* mutant, the mol% amount of MGDG was reduced by 19% compared to wild type and the complemented plants, indicating that *PHD1* is involved in MGDG biosynthesis. In contrast, only a slight decrease (2.5%) in DGDG content was observed in the *phd1*-*1* mutant, demonstrating that *PHD1* may not be required for DGDG synthesis and suggesting that the UDP-Gal substrate for DGDG formation was presumably supplied from the cytosol. Reduced abundance of MGDG in *phd1*-*1* was accompanied by an increase in the abundance of other major membrane lipids such as phosphatidylcholine (PC), phosphatidylglycerol (PG), and phosphatidylinositol (PI), while the mol% levels of sulfoquinovosyldiacylglycerol (SQDG) and phosphatidylethanolamine (PE) were not altered significantly in the *phd1*-*1* mutant (Figure 7A). Because the two galactolipids and SQDG are major components of thylakoid membrane lipids, this result suggests that the mutant had an overall lower amount of chloroplast membrane lipids than wild type plants. Focusing on the exclusive chloroplast lipid MGDG, the fatty acid composition was also investigated (Figure 7B). MGDG of the *phd1*-*1* mutant contained considerably decreased levels of stearic acid (18∶0) compared with the wild type and elevated levels of linoleic acid (18∶2) and linolenic acid (18∶3). The levels of other fatty acids were similar to those observed in wild type plants. Hexadecatrienoic acid (16∶3), which is typically found in the plant prokaryotic pathway, was not detected in all the rice plants, suggesting that rice entirely relies on endoplasmic reticulum (ER)-derived lipids for thylakoid galactoglycerolipid biosynthesis.

Noninvasive chlorophyll fluorescence measurements indicated that the maximum quantum yields for photosystem II photochemistry (*F*_v_/*F*_m_) were similar for *phd1*-*1* and wild type (Table 1). The effective quantum yield of photochemical energy conversion in photosystem II (Φ_PSII_) was slightly but significantly reduced in the mutant (Table 1). Pigment content was also reduced in the *phd1*-*1* mutant (Table 1). Interestingly, chloroplasts of 2-month-old *phd1*-*1* plants were significantly smaller than those of wild type plants (wild type, 5.0±0.4 µm; *phd1*-*1*, 3.0±0.5 µm), and starch grains were also either absent or reduced in size and/or number in the mutant (Figure S7). These data indicated that a reduced amount of galactolipids in chloroplasts and perhaps a smaller size of chloroplasts due to a decrease in membrane lipid content might lead to reduced photosynthetic capability of higher plants.

**Table 1:** Pigment contents (mg·g^−1^ fresh weight) and photosynthetic parameters of wild type, *phd1*-*1*, and *PHD1*-complemented plants.

	Wild type	*phd1*-*1*	*phd1*-*1*+*PHD1*
Chlorophyll *a*	2.50±0.34	1.86±0.36*	2.48±0.37
Chlorophyll *b*	0.96±0.13	0.67±0.12*	0.93±0.18
Chlorophyll *a*+*b*	3.46±0.42	2.53±0.43*	3.41±0.52
Chlorophyll *a*/*b*	2.62±0.37	2.77±0.41	2.66±0.55
Carotenoids	0.33±0.04	0.28±0.05	0.34±0.07
*F*_v_/*F*_m_	0.84±0.01	0.79±0.01	0.83±0.02
Φ_PSII_	0.72±0.01	0.58±0.02*	0.70±0.02

Samples were collected from fully expanded leaves of 4-month-old plants grown in paddy fields. Values represent means (± SD) of six to ten independent determinations.

*: Significant difference between mutant and wild type (*P*<0.05).

### *PHD1* influences the homeostasis of carbon assimilation in leaves

**Figure 8:**
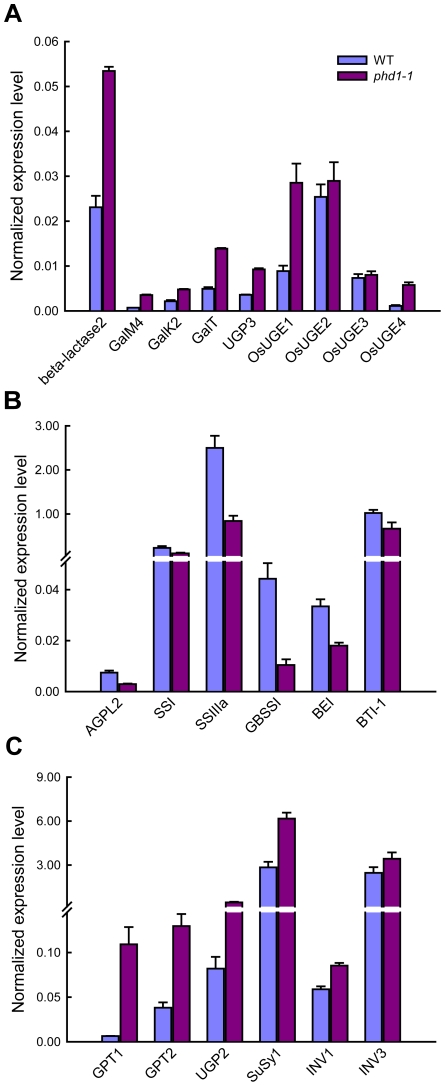
Expression analysis of key genes involved in UDP-Gal biosynthesis and carbohydrate allocation in leaves of *phd1*-*1* plants. (A) The expression of genes involved in the UDP-Gal biosynthesis pathway was upregulated in *phd1-1*. (B) The expression of starch biosynthesis genes was downregulated in *phd1-1*. (C) The expression of sucrose cleavage genes was upregulated in *phd1-1*. Expression values are displayed as the ratio of expression to rice 18S RNA (mean ± SE). All assays were carried out with three biological replicates.

UDP-Gal is the activated form of galactose in biosynthetic reactions, but a galactose salvage pathway exists in eukaryotic organisms. To assess expression of genes involved in the Leloir salvage pathway, the expression levels of three key genes of this pathway, *GalM*, *GalK*, and *GalT*, were analyzed in both *phd1*-*1* and wild type. The expression of all three genes was significantly upregulated in the *phd1*-*1* mutant, suggesting an activation of the whole salvage pathway (Figure 8A). β-Lactase is involved in the generation of free β-D-Gal from polysaccharide breakdown, and UDP-Glc pyrophosphorylase (UGP) catalyzes the formation of UDP-Glc from Glc-1-P. The expression levels of genes encoding β-lactase and UGP3 were also upregulated in *phd1*-*1*. More strikingly, the expression levels of *OsUGE1* and *OsUGE4* encoding for putative cytoplasmic isoforms of UGE in rice were more than two-fold higher in *phd1*-*1* than in wild type plants, indicating an upregulation of *de novo* UDP-Gal biosynthesis in the cytoplasm. These results suggested that *PHD1* may be responsible for a majority of the UGE function in chloroplasts, and appears to be involved in the generation of UDP-Gal from UDP-Glc to supply building blocks for galactolipid biosynthesis required for proper chloroplast membrane composition.

Because the *phd1*-*1* mutant exhibited a dramatic decrease of carbon assimilate levels, we determined whether transcript levels of several key genes involved in the synthesis, transport, and cleavage of starch and sucrose were altered in mature leaves of wild type and *phd1*-*1* plants. Interestingly, while the expressions of starch biosynthesis genes such as *AGPL2*, *SSI*, *SSIIIa*, *GBSS*, *BE*, and *BT1*, were suppressed in the *phd1*-*1* mutant (Figure 8B), expression levels of genes participating in sucrose cleavage, such as *INV1*/*3* and *SuSy1*, were all increased (Figure 8C). Meanwhile, the *GPT* gene encoding a glucose-6-phosphate/phosphate translocator was upregulated in *phd1*-*1*, implying an enhanced export of hexose-phosphates from chloroplasts to the cytosol. In addition, increased expression level of *UGP2*, a gene involved in UDP-Glc synthesis, was correlated with increased UDP-Glc accumulation and a higher UDP-Glc/UDP-Gal ratio in the *phd1*-*1* mutant.

### Overexpression of *PHD1* increases growth rate and grain yield

**Figure 9:**
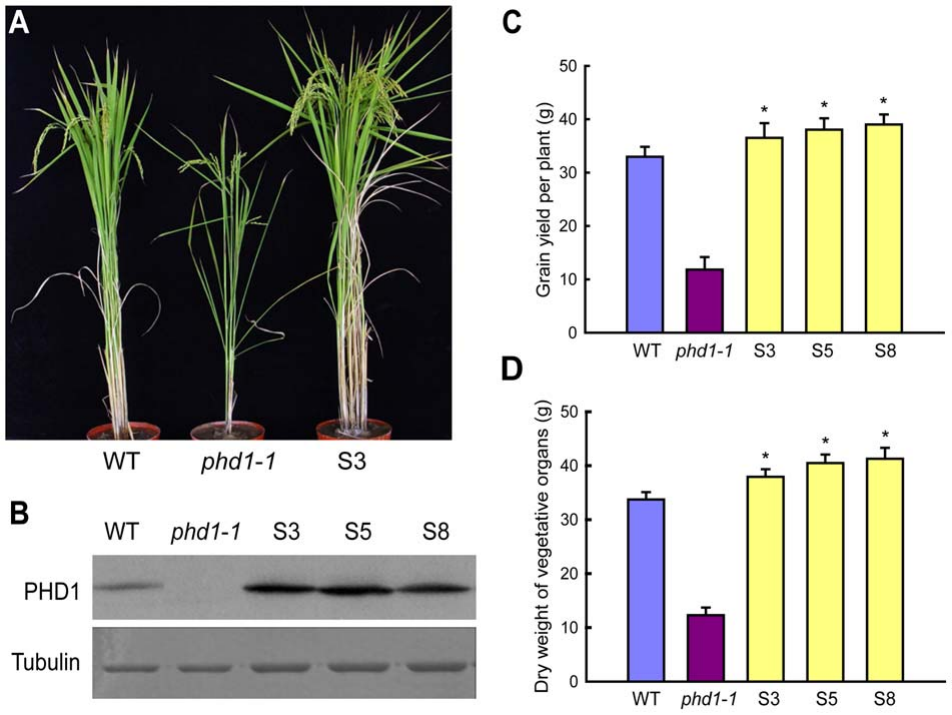
Agricultural traits of transgenic rice lines overexpressing *PHD1*. (A) Phenotypic differences of wild type, *phd1*-*1*, and transgenic line S3 at the grain-filling stage grown in paddy fields. (B) Immunoblot analysis of PHD1 protein expression in wild type, *phd1*-*1*, and *PHD1* overexpressing transgenic lines (S3, S5, and S8). Tubulin is shown as loading control. Increased accumulation of PHD1 protein was observed in transgenic lines. (C, D) Grain yield per plant (C) and dry weight of vegetative organs after harvesting (D) were increased in transgenic plants. Values are means ± SD from at least 30 plants/line. Asterisks indicate a statistically significant difference from the wild type (*P<0.05, Student's *t*-test).

Since a mutation in *PHD1* affected photosynthesis and growth rate, we further investigated whether biomass and grain yield could be improved by *PHD1* overexpression. When grown in paddy fields, transgenic rice plants overexpressing *PHD1* showed a significant increase in tillering (branching) and photosynthetic rate (Figure 9A, Table S1) in lines that overexpressed the PHD1 protein (Figure 9B). Compared to non-transgenic control plants, grain yield per plant of transgenic lines S3, S5, and S8 increased 10.7, 15.5, and 18.3%, respectively (Figure 9C). In addition, the growth rate of transgenic plants accelerated at the seedling stage and dry material accumulation was enhanced 12.5% to 22.4% at the mature stage compared to non-transgenic plants (Figure 9D, Table S1). These results demonstrated that *PHD1* overexpression in rice is positively correlated with an increase in biomass production and grain yield.

## Discussion

**Figure 10:**
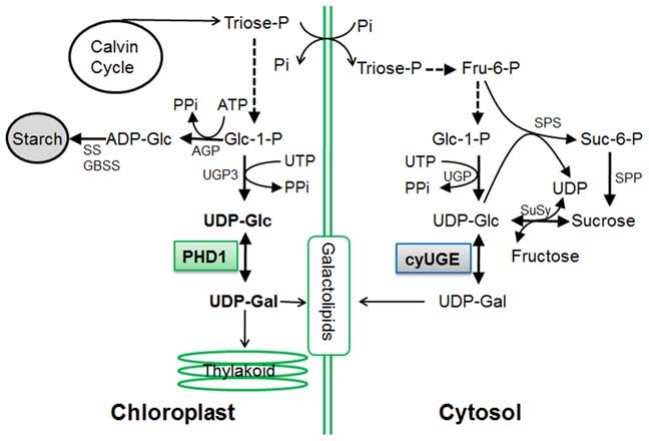
Proposed model for the role of PHD1 in the galactolipid biosynthetic pathway for chloroplast membranes. The substrates of the reaction catalyzed by PHD1 are in bold. AGP, ADP-glucose pyrophosphorylase; SS and GBSS, starch synthases; UGP, UDP-Glc pyrophosphorylase; CyUGE, cytosolic UDP-Glucose 4-epimerase; SPS, sucrose phosphate synthase; SPP, sucrose phosphate phosphatase; SuSy, sucrose synthase; Glc-1-P, glucose-1-phosphate; Pi, inorganic phosphate; PPi, pyrophosphate; ATP, adenosine-triphosphate. Intermediates linked by a single reaction are represented by an unbroken line.

To date all *UGE* genes coding for UDP-Glc epimerases isolated from plants are localized to the cytosol, where their substrates UDP-Glc and UDP-Gal are present at high levels [30]. As a precursor for the synthesis of the galactolipid MGDG in chloroplasts, UDP-Gal is widely assumed to be mobilized from the cytosol, because the UDP-Gal concentration is relatively low within plastids [28] and MGDG synthase (MGD1) is associated with the inner envelope membrane [26], [27]. However, a labeling experiment in which radioactively labeled UDP-Gal was applied to isolated Arabidopsis chloroplasts revealed that radioactivity was not efficiently incorporated into MGDG [23], raising the question of how UDP-Gal is transported into the chloroplasts. In this study, we found that a mutation in *PHD1*, which encodes a novel rice plastidial UGE involved in the biosynthesis of chloroplast galactolipids, lead to disturbed carbon assimilation homeostasis and impaired photosynthetic efficiency. Our work revealed that *PHD1* codes for an active epimerase that is targeted to chloroplasts, and, therefore, that the UDP-Gal substrate for MGDG biosynthesis can be generated *in situ* in chloroplasts (Figure 10). The novel finding that this UGE is chloroplast-targeted was supported by three independent lines of evidence (Figure 5). First, PHD1-GFP fusion products were found exclusively in chloroplasts. Second, Western blot analyses of fractionated chloroplasts showed that PHD1 was highly enriched in the stroma fraction of chloroplasts. And third, immunocytochemistry indicated that PHD1 was concentrated inside the chloroplast stroma, most likely associated with the thylakoid surface. This striking result provides a well-defined genetic and biochemical framework to study the novel functional mechanism of this UGE in plastids, and to evaluate the role of galactolipids in photosynthetic activity of rice.

Of MGDG synthases that are primarily important for thylakoid membrane biogenesis, MGD1 is considered to be the major isoform [24]. In Arabidopsis, two more MGDG synthases, MGD2 and MGD3, are targeted to the outer chloroplast envelope where substrates can be recruited from the cytosol [27]. MGDG generated by them can move from the outer to the inner envelope and to the thylakoids. Here we show that compared to wild type, the relative amount of the major galactolipid MGDG was reduced by 19% in the *phd1*-*1* mutant, whereas that of DGDG was only slightly decreased by 2.5%. We observed a slight increase in the mol% amount of the thylakoid lipid phosphatidylglycerol, which may compensate for a fraction of the galactolipids lost in the *phd1*-*1* mutant. Meanwhile, the relative amount of several extraplastid phospholipids was found to be slightly but significantly higher in the *phd1*-*1* mutant, suggesting that compared to extraplastidic membranes, the overall amount of plastid membranes might have decreased. These results are consistent with the hypothesis that the amounts of glycolipids and phospholipids are reciprocally controlled in plants to maintain a proper balance of lipids in the ER and plastid membrane systems [20], [31]. It has been shown previously that osmotic stress induced variations in membrane fluidity that correlated with the physical properties of membrane lipids [32]. Due to an overabundance of UDP-Glc observed in chloroplasts and entire leaves of the *phd1*-*1* mutant, hyperosmotic stress might occur, and an increased production of 18∶3 could affect hyperosmotic stress tolerance in the mutant chloroplasts. This would be in agreement with earlier observations that transgenic enhancement of fatty acid unsaturation rendered cells and whole plants more tolerant to sorbitol-induced osmotic stress in tobacco [33].

Most galactolipids are restricted to plastid membranes during normal growth and development, however, DGDG can also be found in extraplastidic membranes following phosphate (Pi) starvation [34], [35]. Importantly, x-ray crystallographic analyses of photosynthetic proteins in cyanobacteria revealed that MGDG is associated with the core of the reaction centers of both photosystems I and II (PSI and PSII) [36], [37], which suggest that these lipids are required not only as bulk constituents of photosynthetic membranes, but also for the photosynthetic reaction itself. Consistent with this, we found that the effective quantum yield of photochemical energy conversion in photosystem II (Φ_PSII_) was reduced in the *phd1*-*1* mutant. Seedlings lacking MGDG were previously shown to have disrupted photosynthetic membranes, leading to a complete impairment of photosynthetic ability and photoautotrophic growth [22], [24]. In agreement with this, a possible reduction of thylakoid membrane amount and a changed galactolipid to phospholipid ratio in chloroplast membranes in the *phd1*-*1* mutant might have led to the dramatic phenotype of retarded growth, reduced photosynthetic capability, and decreased photoassimilate accumulation. Taken together, this strongly suggests that the stunted growth phenotype of *phd1*-*1* mutants is due to an insufficient provision or slower production of membrane building blocks to support chloroplast proliferation during plant growth, which is also consistent with the reduced numbers of thylakoid stacks and sizes of chloroplasts observed in mutant plants.

In plants, starch acts as a depository for reduced carbon produced in leaves during the day, and as a supply of chemical energy and anabolic source molecules during the night [38]. Pyrophosphate (PPi) is produced during the upregulation of *UGP3* (Figure 10), and hydrolyzed by very high pyrophosphatase (PPase) activity in plastids [39]. Moreover, inorganic phosphate (Pi) released during PPi hydrolyzation is an inhibitor for key regulatory starch biosynthesis enzymes such as AGP [40]. In the *phd1*-*1* mutant, expression levels of starch biosynthesis genes such as *AGP*, *SS*, *GBSS*, and *BE*, were significantly downregulated in source leaves, leading to a sharp decrease of starch content. However, the reduced starch did not result in increased sucrose levels, because activation of sucrose cleavage genes *SuSy1* and *INV1*/*3* resulted in reduced sucrose and increased hexose-phosphate and UDP-Glc levels. Therefore, sucrose as the main transport form of photoassimilate produced in source organs was not able to export efficiently to the sink organs. Moreover, a large amount of UDP-Glc catalyzed by SuSy1 or UGP2 would be converted to UDP-Gal by cytosolic OsUGE1/4 and transported into chloroplast as galactosyl donors of chloroplast glycolipids to compensate for the loss of PHD1 activity in the *phd1*-*1* mutant. In contrast, *PHD1* overexpression in rice, which enhanced PHD1 activity in chloroplasts (Figure S5), might increase the relative amount of MGDG and increase the effective quantum yield of photochemical energy conversion in thylakoid membranes, resulting in increased photosynthetic efficiency and growth rate, implicating a key role of PHD1 for the photosynthetic system in rice. These improvements of both biomass production and grain yield have significant economic implications in both traditional crop improvement and bioenergy crop production.

## Materials and Methods

### Plant material and growth conditions

The rice (*Oryza sativa* L.) *phd1* mutant is in the Nipponbare (ssp *japonica*) background. F2 mapping populations were generated from a cross between the rice *phd1* mutant and MH63 (ssp *indica*). Rice plants were cultivated in the experimental station of the Institute of Genetics and Developmental Biology (IGDB) in Beijing in natural growing seasons. For analysis of diurnal changes of starch and sugars, rice plants were kept in a growth chamber at 28°C and 70% relative humidity under a photoperiod of 12 h light/12 h darkness, with a light intensity of 200 µmol quanta m^−2^ s^−1^.

### Map-based cloning

Genomic DNA was isolated from seedlings of the selected plants with the mutant phenotype. For fine mapping of *PHD1*, STS markers were generated based on the polymorphisms between Nipponbare and MH63. The molecular lesion of *phd1*-*1* was identified by PCR amplification of the *PHD1* genomic region from wild type and *phd1*-*1* mutant plants and comparison of their sequences. The candidate gene was mapped between the 2 new STS markers S221 (5′-AGAGCTAGGGGGTAAAAA-3′ and 5′-GTGCAGAACAGTGGAATG-3′) and S246 (5′-AACCCTATCCTTCCTCACCA-3′ and 5′-TTGTCCCTCCGCCTGCTTCC-3′).

### Database search and phylogenetic analysis

PHD1 homologs were detected by BLASTp using the entire amino acid sequence of PHD1 as a query in the National Center for Biotechnology Information database (http://www.ncbi.nlm.nih.gov/BLAST). Multiple alignment of the homologs was performed by Clustal X version 2.0 with the default parameters [41] and manually adjusted. For constructing phylogenetic trees, the neighbor-joining method of the MEGA 4.1 software [42] was used, and a bootstrap analysis with 1 000 replicates was performed to test the confidence of topology.

### Generation of transgenic rice plants

The BAC clone BAC53 containing the entire *PHD1* fragment was digested with *Sac* I and *Pst* I to generate a 7.96 kb genomic DNA fragment. The DNA fragment was ligated to the *Sac* I and *Pst* I digested pCAMBIA1300 vector (CAMBIA), to generate the pSCL construct for complementation analysis. The full-length *PHD1* cDNA was PCR amplified using primers 5′-GATCCGATCCCCTCACCTC-3′ and 5′- TTCTCTGGCCGAAACCATT-3′, and subcloned into the pCAMBIA2300-35S binary vector, between the cauliflower mosaic virus 35S promoter and nopaline synthase (nos) terminator, to generate the pSOL construct for overexpression analysis. Transgenic rice plants were generated according to *Agrobacterium tumefaciens*-mediated transformation methods [43], [44]. The transgenic plants were then transferred to the field at the IGDB experimental station for normal growth and seed harvesting.

### Protein and RNA analyses

*PHD1* cDNA was amplified by primer sets 5′-TGATGATACAGGGGTCAAGATG-3′ and 5′-ACTGTCAAGACCAAGGAATTCT-3′ and cloned into the *Xma* I and *Xho* I sites of pGEX-4T-1 (GE Healthcare Life Sciences) and expressed in *E. coli* strain BL21 (DE3). Recombinant PHD1 protein was affinity-purified through glutathione Sepharose resin (Amersham Pharmacia Biotech) and used for antibody production [45].

Total RNA was prepared with an RNeasy kit (Qiagen). In the RNA gel blot analysis, 5 µg of total RNA was electrophoresed on a 1.2% (w/v) agarose gel and transferred to a nylon membrane, and mRNA was detected by a digoxigenin labeling system (Roche Diagnostics). For quantitative RT-PCR, 15 ng of cDNA and SYBR Green SuperMix (Bio-Rad) were used in 15 µL qRT-PCR reactions with a CFX96 96-well real-time PCR detection system (Bio-Rad) and CFX96 software to calculate threshold cycle values, and rice 18S ribosome RNA was used as an internal control. Oligonucleotide primers are given in Table S2. The 2^−ΔΔC^_T_ method was adopted to calculate the relative expression levels for the *phd1* and wild type samples, and a two-tailed *t* test used to compare the ratios and determine statistical significance [46].

### Histological analysis and mRNA *in situ* hybridization

Freshly collected rice tissues were fixed in FAA solution (50% ethanol, 5% acetic acid, 3.8% formaldehyde) at 4°C overnight, dehydrated with ethanol solution from 50% to 100%, cleaned by a series of xylene washes from 25% to 100%, and embedded in paraffin (Paraplast Plus, Sigma-Aldrich) at 54–56°C as described in [47]. 8 to 12 µm sections were cut with a microtome (Leica RM2265), and mounted on RNase-free glass slides and photographed.

RNA *in situ* hybridization was performed as described previously with minor modification [48]. Briefly, the 420-bp region of *PHD1* was amplified by gene-specific primers with T7 or SP6 promoters 5′-*TAATACGACTCACTATAGGG*CCCCTTCTCCGTCAACCT-3′ and 5′-AACGAAAGAGCCTTCACCA-3′ or 5′-CCCCTTCTCCGTCAACCT-3′ and 5′-*ATTTAGGTGACACTATAG*AACGAAAGAGCCTTCACCA-3′ in front of the reverse primer (for making anti-sense probe) or forward primer (for making sense probe). Digoxigenin-labeled RNA probes were prepared using a DIG Northern Starter Kit (Cat. No. 2039672, Roche) according to the manufacturer's instructions. The hybridization signals were observed using bright field imaging with a microscope (Olympus BX51) and photographed with a Micro Color CCD camera (DVC Co. Austin, USA).

### Transient expression assay in rice protoplast cells

A binary vector containing GFP fused with full-length *PHD1* was constructed as follows. The PCR product amplified with primers 5′-ACCTCCGTCCCTGCTTCCTC-3′ and 5′-GGGCTCCCAACCAATCTCA-3′ was subcloned into the CaMV 35S::GFP vector to generate CaMV 35S::*PHD1*-GFP. The binary vector was transformed into rice protoplasts using the polyethylene glycol method [49]. After overnight incubation in the dark, the protoplasts expressing GFP were imaged by a confocal laser scanning microscope (LSM510, Zeiss, Germany) using 488 nm excitation and 500–530 nm emission pass-filters. Chlorophyll autofluorescence was detected with 570 nm excitation and 640 nm emission pass-filter [50]. Composite figures were prepared using Zeiss LSM Image Browser software.

### Cloning and expression of recombinant *PHD1* in yeast

*PHD1* and its derivative cDNAs were amplified by PCR using the primers 5′- ATGATACAGGGGTCAAGATGG-3′ and 5′-ACTGTCAAGACCAAGGAATTCT -3′, and inserted into the vector pDBLeu (Invitrogen). The Euroscarf *S. cerevisiae* strain BY4742 (*Mat*α *his3*Δ*1 leu2*Δ*0 lys2*Δ*0 ura3*Δ*0 gal10*::*kanMX4*) was transformed using a lithium acetate procedure and tested on 1% (w/v) galactose medium (1% (w/v) yeast extract (Duchefa), 2% (w/v) Bacto-peptone (BD Bio- sciences), 1% (w/v) galactose (Sigma), 2% (w/v) Micro agar (Duchefa)).

### Extraction and measurement of carbohydrates

Individual samples (leaves of circa 500 mg fresh weight) were harvested and frozen rapidly in liquid N_2_. The frozen samples were homogenized and extracted with perchloric acid. Glucose, fructose, sucrose, and starch were measured enzymatically for the neutralized supernatant (sugars) and the insoluble pellet (starch) [51]. Determination of UDP-Glc and UDP-Gal were performed as described [11].

### Lipid analysis

Total lipids were extracted from 2-month-old *phd1*-*1*, wild type, and the *PHD1*-complemented plants as described [52]. For quantitative analysis, individual lipids were separated by two-dimensional thin-layer chromatography and used to prepare fatty acid methyl esters. The methyl esters were quantified by gas-liquid chromatography as described [53]. A 1 µl sample was applied for GC-MS (Agilent 7890A GC coupled to 5975C MS) analysis at a 10∶1 split ratio. The GC-MS program started with 80°C for 1 min, then ramped at 8°C/min to 300°C and held for 5 min; injector and inlet temperatures were set at 250°C and 280°C, respectively. Separation was performed on a HP-5 MS column (30 m×0.25 mm×0.25 µm) with a constant flow of 1.1 ml/min helium. The MS scan range was from 50 to 500 m/z. The quantification of fatty acid methyl esters was performed by the external standard method.

### Assay of UGE activity

UGE activity was measured using a NADH-coupled assay developed by Wilson and Hogness [54] with some minor modifications. The 1 ml assay mixture consisted of 100 mM glycine buffer (pH 8.7), 1 mM β-NAD^+^ (Sigma), and 0.8 mM UDP-Gal (Sigma). The reaction was started by adding 10 µl of epimerase (140 µg/ml) in 50 mM Tris·Cl (pH 7.6), 1% (w/v) bovine serum albumin, 1 mM dithiothreitol, 1 mM EDTA, and 1 mM β-NAD^+^, and stopped by incubation for 10 min at 100°C. The UDP-glucose produced was determined by addition of 0.04 unit of bovine UDP-glucose dehydrogenase (Calbiochem) and incubation for 10 min at 30°C, and the increase in absorbance due to NADH formation was then measured at 340 nm. *K*_m_ values were determined by varying the UDP-Gal concentration between 0.4 mM and 3.2 mM. The experiment was conducted in triplicate.

### Purification of chloroplasts and chloroplast subfractions from rice

All isolation procedures were carried out at 4°C. Batches of 50 g rice leaves were cut to little pieces and homogenized in 250 ml of isolation buffer (50 mM HEPES/KOH, pH 7.8, 0.33 M sorbitol, 2 mM EDTA, 1 mM MgCl_2_, 1 mM MnCl_2_, 0.1 M Na-ascorbate, 0.2% (w/v) bovine serum albumin) using a Waring blender. The chloroplast suspension was passed through four layers of Miracloth and centrifuged at 4 000 *g* for 4 min. The pellet was gently suspended in the isolation buffer and layered onto a discontinuous density gradient consisting of 10, 40, and 80% (v/v) Percoll in the isolation buffer. The gradient was centrifuged at 8 000 *g* for 10 min. Intact chloroplasts distributed around the 40/80% Percoll interface were isolated and reapplied to the Percoll gradient centrifugation.

Chloroplasts were lysed by resuspension to 0.5 mg chlorophyll ml^−1^ in 10 mM HEPES/KOH (pH 8.0), 5 mM MgCl_2_, for 20 min on ice, and the lysate was fractionated into envelope, stroma, and thylakoids by differential centrifugation as described by Skalitzky et al [55]. All solutions contained a cocktail of protease inhibitors. To verify recovery and purity of the sucrose density fractions, several antibodies against specific marker proteins were used: Tic40 was used as an envelope marker, RbcL as a stromal marker, and PsbA as a thylakoid membrane marker.

### Immunocytochemistry

Immunoelectron microscopy experiments were carried out as previously described [56]. Briefly, nickel grids carrying ultrathin leaf sections prepared from two-week-old wild type seedlings were sequentially floated in 0.01 M sodium phosphate buffer (PBS, pH 7.2) containing 5% (w/v) bovine serum albumin (BSA) for 5 min, then for 1 h at 37°C in PBS containing diluted anti-PHD1 antibody. After several washes in PBS, ultrathin sections were incubated for 1 h at 37°C in PBS containing goat anti-rabbit IgG antibody conjugated to 10-nm colloidal gold (1∶40, Sigma-Aldrich, St. Louis, MO, USA). After 5 washes with PBS, ultrathin sections were washed with distilled water, air dried, counterstained with 2% uranyl acetate, and examined with a FEI Tecnai G^2^ 20 transmission electron microscopy at an accelerating voltage of 120 kV. Negative controls were performed using the same procedure with the exception of substituting the anti-PHD1 antibody with preimmune serum.

## Supporting Information

Figure S1 Diurnal changes in hexose concentration of *phd1*-*1* and WT. Mature leaves of individual wild type (• black symbols with solid lines) and *phd1-1* (○ empty symbols with broken lines) plants were harvested and immediately frozen in liquid N_2_. Each point is the mean ± standard deviation from ten replicate samples.
(TIF)

Figure S2 *PHD1* transcript levels in wild type, three allelic *phd1* mutants, and one overexpression line. The equal abundance of RNA among samples was confirmed by RT-PCR detection of *ACTIN1* transcripts. *phd1*-*1* to -*3*, three allelic *phd1* mutant lines; S3-1, *PHD1* overexpressing transgenic line.
(TIF)

Figure S3 Protein structure of PHD1 and comparison of the conserved regions of seventeen PHD1 homolog sequences from green plants. (A) Schematic representation of the PHD1 protein structure. Regions of the putative chloroplast transit peptide (cTP) and the nucleoside-diphosphate-sugar epimerase (WcaG) consensus motif (COG0541) are shown in patterned boxes. (B) Amino acid sequences were searched using BLASTP and aligned using CLUSTALW. Identical amino acid residues are boxed, and similar residues are shaded. The red bar indicates the conserved motif GXGXXG (NAD^+^-binding), and catalytic amino acid residues of the active site are boxed in red. PHD1: Os01g0367100.
(TIF)

Figure S4 Biochemical function and genetic complementation assay of PHD1. (A) UGE activity assay of PHD1 *in vitro*. Lineweaver-Burk plots of purified recombinant PHD1 UGE activity at 30°C (▪) and at 37°C (□). Values are the means ± SDs. (B) PHD1 can complement a *S. cerevisiae gal10* mutant. A yeast *gal10* mutant strain was transformed with plasmids containing *PHD1* cDNAs, and grown on either glucose or galactose medium.
(TIF)

Figure S5 UGE activity in isolated chloroplasts of wild type, *phd1*-*1*, and *PHD1*-overexpressing plants. Intact chloroplasts were isolated from the leaves of wild type, *phd1*-*1*, and *PHD1*-overexpressing transgenic lines (S3, S5, and S8) by step-wise density gradient centrifugation, and UGE activity was determined as described in Materials and Methods. Values are the mean of three experiments ± SDs. Asterisks indicate a statistically significant difference from the wild type (*P<0.05, Student's *t*-test).
(TIF)

Figure S6 UDP-Glc and UDP-Gal contents in leaves of wild type, *phd1*-*1*, and *PHD1*-complemented plants. The values represent the means ± SE of six independent repeats.
(TIF)

Figure S7 Starch accumulation and chloroplast ultrastructures in leaves of wild type (A) and *phd1*-*1* (B) plants. Leaf samples were harvested at 9 h under a 12-h photoperiod and prepared for TEM. Bars = 1 µm.
(TIF)

Table S1 Characterization of biomass and photosynthetic rate of wild type (Nipponbare) and *PHD1*-overexpressing plants.
(DOC)

Table S2 Oligonucleotides used in this study.
(DOC)
