# Supplementary figures and images for: A Rice Plastidial Nucleotide Sugar Epimerase Is Involved in Galactolipid Biosynthesis and Improves Photosynthetic Efficiency
Source: PLoS Genet. 2011 Jul 28;7(7):e1002196. doi: 10.1371/journal.pgen.1002196 (PMC3145628; doi:10.1371/journal.pgen.1002196)

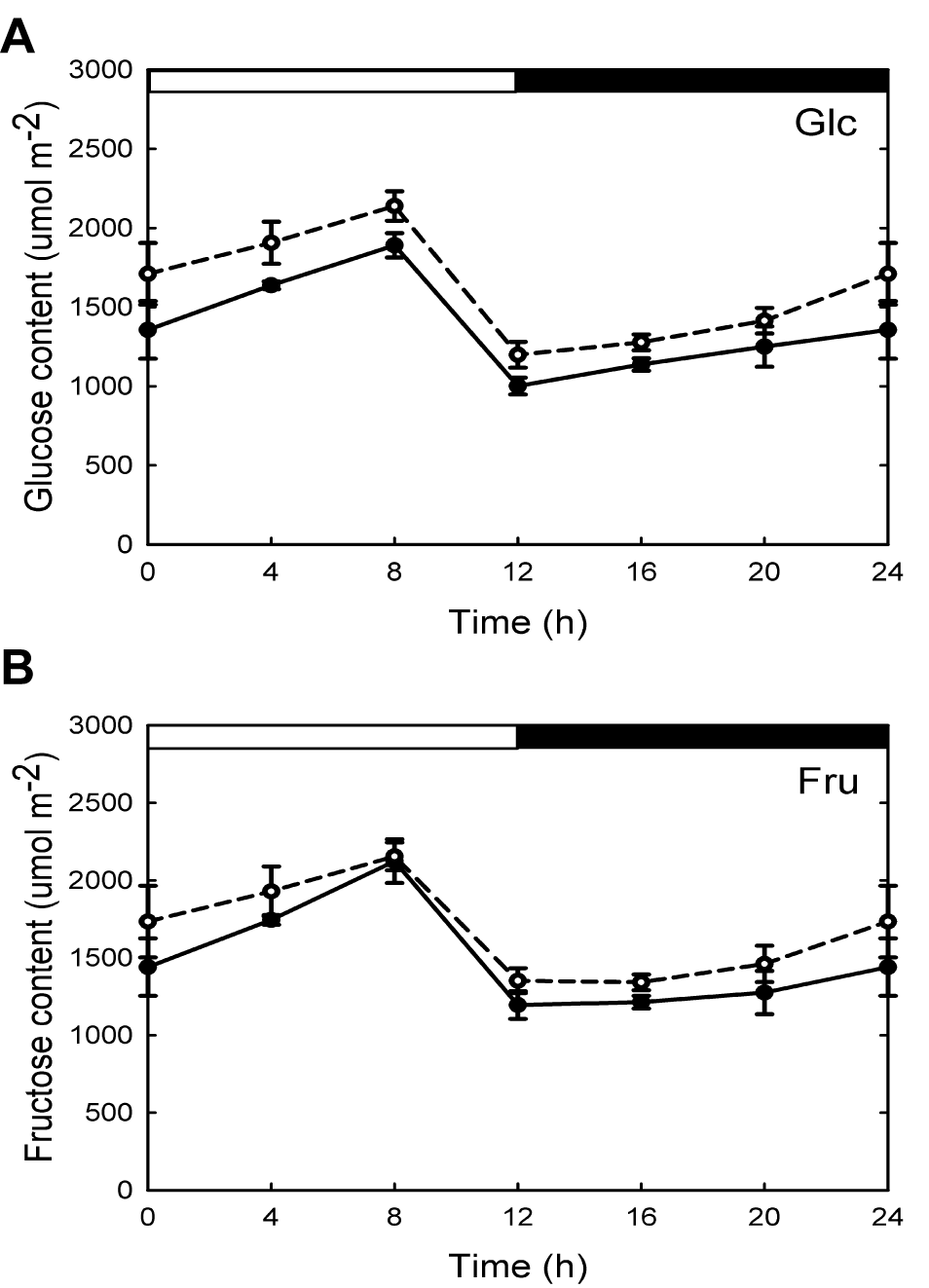

Supplement: Figure S1 — Diurnal changes in hexose concentration of phd1-1 and WT. Mature leaves of individual wild type (• black symbols with solid lines) and phd1-1 (○ empty symbols with broken lines) plants were harvested and immediately frozen in liquid N2. Each point is the mean ± standard deviation from ten replicate samples. (TIF) [file pgen.1002196.s001.tif]

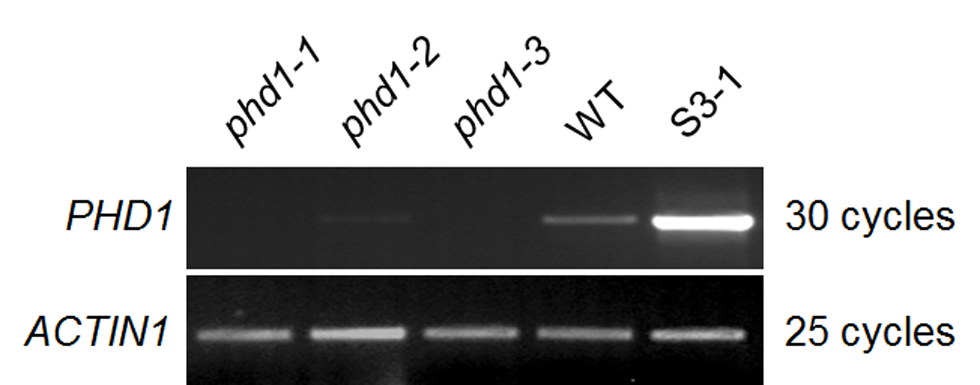

Supplement: Figure S2 — PHD1 transcript levels in wild type, three allelic phd1 mutants, and one overexpression line. The equal abundance of RNA among samples was confirmed by RT-PCR detection of ACTIN1 transcripts. phd1-1 to -3, three allelic phd1 mutant lines; S3-1, PHD1 overexpressing transgenic line. (TIF) [file pgen.1002196.s002.tif]

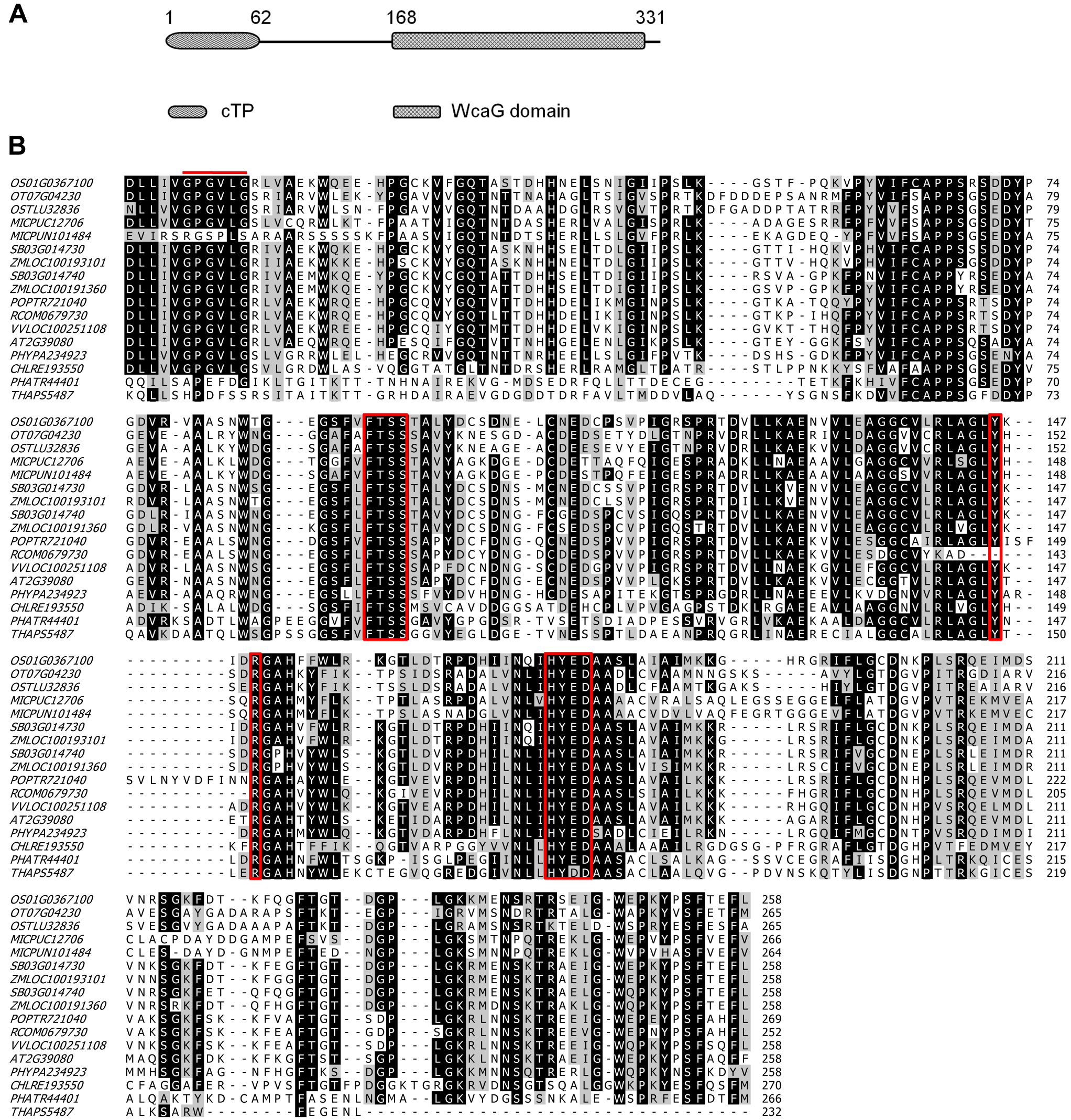

Supplement: Figure S3 — Protein structure of PHD1 and comparison of the conserved regions of seventeen PHD1 homolog sequences from green plants. (A) Schematic representation of the PHD1 protein structure. Regions of the putative chloroplast transit peptide (cTP) and the nucleoside-diphosphate-sugar epimerase (WcaG) consensus motif (COG0541) are shown in patterned boxes. (B) Amino acid sequences were searched using BLASTP and aligned using CLUSTALW. Identical amino acid residues are boxed, and similar residues are shaded. The red bar indicates the conserved motif GXGXXG (NAD+-binding), and catalytic amino acid residues of the active site are boxed in red. PHD1: Os01g0367100. (TIF) [file pgen.1002196.s003.tif]

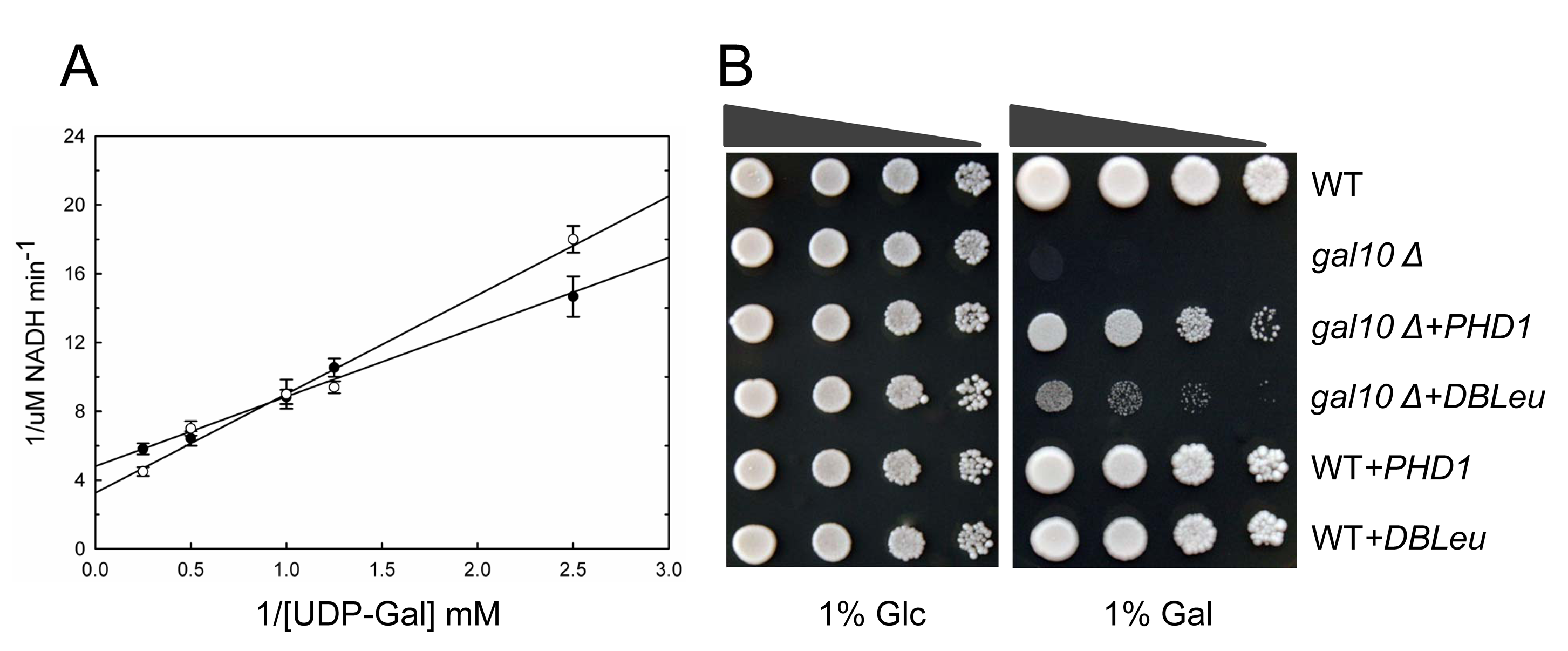

Supplement: Figure S4 — Biochemical function and genetic complementation assay of PHD1. (A) UGE activity assay of PHD1 in vitro. Lineweaver-Burk plots of purified recombinant PHD1 UGE activity at 30°C (▪) and at 37°C (□). Values are the means ± SDs. (B) PHD1 can complement a S. cerevisiae gal10 mutant. A yeast gal10 mutant strain was transformed with plasmids containing PHD1 cDNAs, and grown on either glucose or galactose medium. (TIF) [file pgen.1002196.s004.tif]

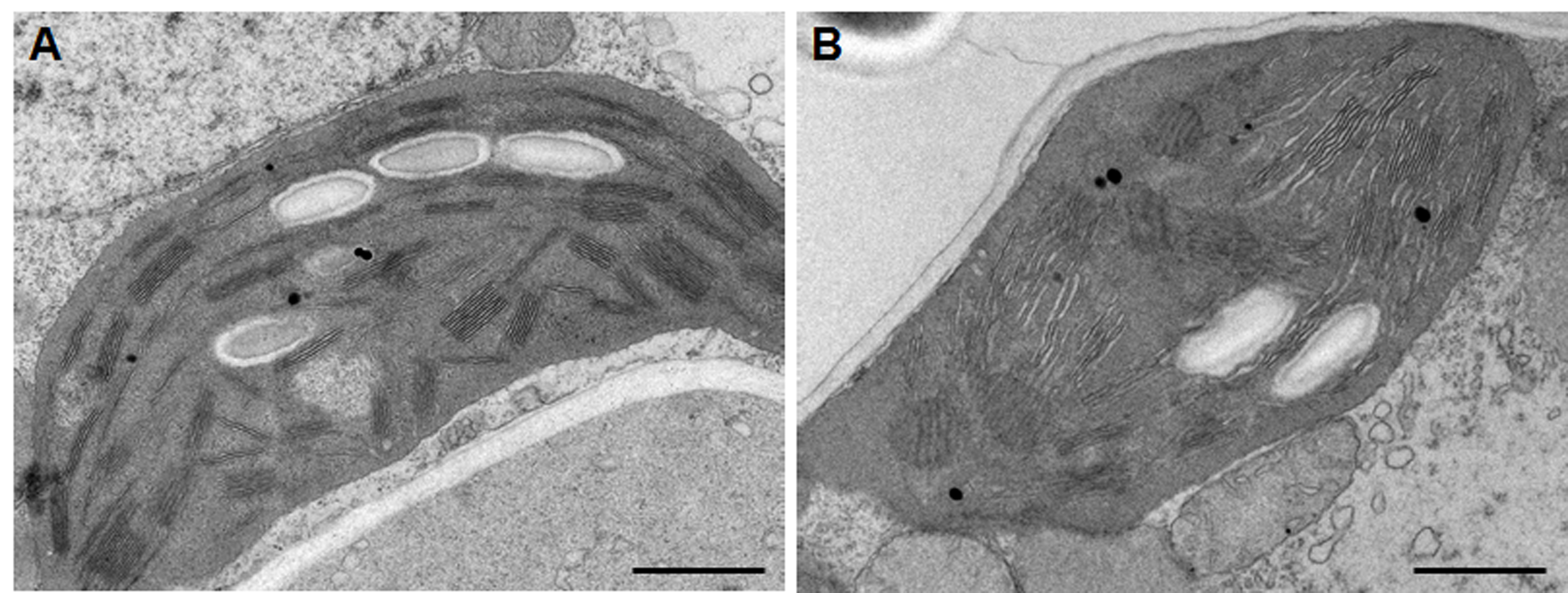

Supplement: Figure S7 — Starch accumulation and chloroplast ultrastructures in leaves of wild type (A) and phd1-1 (B) plants. Leaf samples were harvested at 9 h under a 12-h photoperiod and prepared for TEM. Bars = 1 µm. (TIF) [file pgen.1002196.s007.tif]
