# Supplementary material for: A Rice Plastidial Nucleotide Sugar Epimerase Is Involved in Galactolipid Biosynthesis and Improves Photosynthetic Efficiency
Source: PLoS Genet. 2011 Jul 28;7(7):e1002196. doi: 10.1371/journal.pgen.1002196 (PMC3145628; doi:10.1371/journal.pgen.1002196)
Supplement: Table S1 — Characterization of biomass and photosynthetic rate of wild type (Nipponbare) and PHD1-overexpressing plants. (DOC) [file pgen.1002196.s008.doc]

**Table S1.** Characterization of biomass and photosynthetic rate of wild type (Nipponbare) and *PHD1*-overexpressing plants.

|  | **WT** | **S3** | **S5** | **S8** |
| --- | --- | --- | --- | --- |
| Shoot height (cm)^a^ | 15.32 ± 0.15 | 18.64 ± 0.18^*^ | 19.21 ± 0.33^*^ | 19.64 ± 0.37^*^ |
| Shoot mass (mg)^a^ | 54.73 ± 1.02 | 63.45 ± 1.13^*^ | 66.55 ± 1.17^*^ | 65.91 ± 1.20^*^ |
| NPR (500 μmol·m^-2^·s^-1^)^b^ | 8.31 ± 0.24 | 9.65 ± 0. 53^*^ | 10.12 ± 0.37^*^ | 10.39 ± 0.59^*^ |
| NPR (2000 μmol·m^-2^·s^-1^)^b^ | 14.50 ± 0.99 | 15.78 ± 0.86 | 16.12 ± 1.28 | 16.36 ± 1.36 |

S3, S5, and S8 represent three independent *PHD1*-overexpressing transgenic lines. ^a^20-day-old rice seedlings; ^b^NPR, rate of net photosynthesis (μmol·CO_2_·m^-2^·s^-1^) measured at the heading stage; Values are means ± SD from at least 30 plants/line, ^*^ Significant difference (*P* < 0.05).
