# Supplementary material for: A Rice Plastidial Nucleotide Sugar Epimerase Is Involved in Galactolipid Biosynthesis and Improves Photosynthetic Efficiency
Source: PLoS Genet. 2011 Jul 28;7(7):e1002196. doi: 10.1371/journal.pgen.1002196 (PMC3145628; doi:10.1371/journal.pgen.1002196)
Supplement: Table S2 — Oligonucleotides used in this study. (DOC) [file pgen.1002196.s009.doc]

**Table S2.** Oligonucleotides used in this study.

| **Name** | **Sequence (5’-3’)** |
| --- | --- |
| *β-lactase2* | GTCGTGCCATGACATCTACCA |
|  | CTGCTTTATTGCCTCACTTGC |
| *GalM4* | CGTCGTGCTTCCTGACTCCA |
|  | CCTCCACCAACATGCTCCTTC |
| *GalK2* | ACATAACCTACCGAAGAAGAGTGG |
|  | TCACAGCCTGAAGCACATAAAA |
| *GalT* | GGATACGGCACTGGATCTTGG |
|  | TTGAATGGAGGGTCGTTGAGC |
| *OsUGE1* | TACTGCTCCGATACTTCAACCC |
|  | CCATCCGCTAGATCAACAACAT |
| *OsUGE2* | CCAAGACGCCCTGGTGATGC |
|  | TTCGCTTTCCAGTTGAGTTCCTTC |
| *OsUGE3* | TCGCTACTCCTGACATTGGTT |
|  | TGATCGCCCTAATTCTGCTC |
| *OsUGE4* | TGGAACAGGAAAGGGAACATC |
|  | TCGTGGACCAATAACCAAAGG |
| *UGP3* | GCCAGAACAAACCCATCAAAC |
|  | GTAACTCCAGAGCCGAACCAG |
| *AGPL2* | ATAATCTCCGATGGCTGTTC |
|  | TCCAGACCTTATGTAGTATCCC |
| *SSI* | GGGCCTTCATGGATCAACC |
|  | CCGCTTCAAGCATCCTCATC |
| *SSIIIa* | GCCTGCCCTGGACTACATTG |
|  | GCAAACATATGTACACGGTTCTGG |
| *GBSSI* | AACGTGGCTGCTCCTTGAA |
|  | TTGGCAATAAGCCACACACA |
| *BEI* | TGGCCATGGAAGAGTTGGC |
|  | CAGAAGCAACTGCTCCACC |
| *BTI*-*1* | GAAGTCCTTGAGCCGTCCTG |
|  | AAGTCCCTTGATGCCCTCCT |
| *GPT1* | AGAAGGGATCCAGATGAAGAA |
|  | AACAAGAAACGAGCAACATAGA |
| *GPT2* | GCCTCCATCATCATCTTCCA |
|  | ATTGTTACATCCCGAGCACC |
| *UGP2* | GCCAGAACAAACCCATCAAAC |
|  | GTAACTCCAGAGCCGAACCAG |
| *SuSy1* | GCTTCCACATTGACCCATAC |
|  | CTTGAGGGCATACAGCATCT |
| *INV1* | CACGACGCAGTGATCTGAGG |
|  | GATGAAACGCAGGGAATACG |
| *INV3* | GACATCGTCAAGAGGGTCG |
|  | CCATCCATGATCCATCATCC |
| *ACTIN1* | ACATCGCCCTGGACTATGACCA |
|  | GTCGTACTCAGCCTTGGCAAT |
